# Concerned Journals, Editors And ICMJE

**DOI:** 10.4103/0973-1229.32152

**Published:** 2007

This section will detail some important initiatives taken by journal editors in writing about industry influence on academia in particular and medicine in general. It will also look into the Revised Guidelines of the ICMJE vis-á-vis issues related to industry-sponsored research.

## Journals And Editors

In the last chapter, we saw how conflict of interest related to individuals and institutions are discussed and resolved by AAMC. Conflict of interest because of industry’s increasing clout has also engaged the attention of editors of important journals and some significant editorials in this connection are worth a close study by all concerned researchers and institutions, for they reflect reasoned responses to disturbing developments. The titles themselves reflect their concern:

### Is academic medicine for sale? (Angell, * N Engl J Med*, 2000)

Angell asks a piquant question of academic medical institutions:

‘Academic medical institutions are themselves growing increasingly beholden to industry. How can they justify rigorous conflict-of-interest policies for individual researchers when their own ties are so extensive?’

Answering as to why Academia-industry connect flourishes, she says:

‘Some academic institutions have entered into partnerships with drug companies to set up research centers and teaching programs in which students and faculty members essentially carry out industry research. Both sides see great benefit in this arrangement. For financially struggling medical centers, it means cash. For the companies that make the drugs and devices, it means access to research talent, as well as affiliation with a prestigious “brand.”

Asking herself the question: ‘Why shouldn’t clinical researchers have close ties to industry?’she answers it decisively tilts academia’s inclinations to serve industry interests in various ways, rather than that of biomedical advance:

‘One obvious concern is that these ties will bias research, both the kind of work that is done and the way it is reported. Researchers might undertake studies on the basis of whether they can get industry funding, not whether the studies are scientifically important. That would mean more research on drugs and devices and less designed to gain insights into the causes and mechanisms of disease. It would also skew research toward finding trivial differences between drugs, because those differences can be exploited for marketing. Of even greater concern is the possibility that financial ties may influence the outcome of research studies.’

Lamenting that business concerns of industry might supercede the mission of a medical college, she is forthright in condemning blurring of boundaries between them:

‘When the boundaries between industry and academic medicine become as blurred as they now are, the business goals of industry influence the mission of the medical schools in multiple ways’

And the issue of conflict of commitment surfaces in such faculty members:

‘Finally, there is the issue of conflicts of commitment. Faculty members who do extensive work for industry may be distracted from their commitment to the school’s educational mission.’

The edit recommends an arms length approach as to standards and ethical norms between academia and industry, even as there is increased cooperation, simply because their goals and mission are different:

‘All of this is not to gainsay the importance of the spectacular advances in therapy and diagnosis made possible by new drugs and devices. Nor is it to deny the value of cooperation between academia and industry. But that cooperation should be at arm’s length, with both sides maintaining their own standards and ethical norms. The incentives of the marketplace should not become woven into the fabric of academic medicine. We need to remember that for-profit businesses are pledged to increase the value of their investors’stock. That is a very different goal from the mission of medical schools.’

Answering whether conflict of interest should be softened or made more stringent in the light of increased academia-industry connect, she advocates the latter. She urges major medical schools like Harvard to be unrelenting in its pursuit. Consensus of strict norms between major schools will stem faculty drain to more lax schools:

‘What needs to be done - or undone? Softening its conflict-of-interest guidelines is exactly the wrong thing for Harvard Medical School to do. Instead, it should seek to encourage other institutions to adopt stronger ones. If there were general agreement among the major medical schools on uniform and rigorous rules, the concern about losing faculty to more lax schools - and the consequent race to the bottom - would end.’

Detailing which ties need to be eschewed, she lists equity, most writing/speaking assignments and such lucrative incomes as are greater than salaries, for such income is impossible without shortchanging institutional interests. Similarly, sponsored trips, meals, gifts, conferences, grants and such other expenses simply add on to drug/device MRP:

‘Certain financial ties should be prohibited altogether, including equity interest and many of the writing and speaking arrangements. Rules regarding conflicts of commitment should also be enforced. It is difficult to believe that full-time faculty members can generate outside income greater than their salaries without shortchanging their institutions and students.It is well to remember that the costs of the industry-sponsored trips, meals, gifts, conferences and symposiums and the honorariums, consulting fees and research grants are simply added to the prices of drugs and devices.’

Warning that public trust and goodwill for medicine is waning, she cautions that when in difficulty, the public will have little sympathy for its difficulties if academia is perceived to have cosy relations with industry that compromise its integrity and help escalate prices:

‘In these difficult times, academic medicine depends more than ever on the public’s trust and goodwill. If the public begins to perceive academic medical institutions and clinical researchers as gaining inappropriately from cozy relations with industry - relations that create conflicts of interest and contribute to rising drug prices - there will be little sympathy for their difficulties.’

Academia is cautioned to avoid becoming industry henchmen:

‘Academic institutions and their clinical faculty members must take care not to be open to the charge that they are for sale.’

We have discussed this edit in some detail for it raises some fundamental issues which academia and related industry need to seriously ponder over.

### The controlling interests of research (Editorial, *CMAJ*, 2002a)

This editorial accepts that marriage of academic research with private funding is necessary not because resistance is futile but because it has been beneficial to academia; and public funds, by themselves, cannot do the job, especially in technology intensive research fields. However, such partnerships need to be managed better:

‘We must have partnerships, but we have to manage them better. not because resistance is futile (it may be), but because partnerships with industry have been beneficial.’

However, it warns that partnerships should be so carefully structured that nothing which compromises the rights of human research subjects or the intellectual freedom of researchers becomes acceptable:

‘But these partnerships must be carefully structured to protect the rights of research subjects and the intellectual freedom of scientists’.

### The invisible hand of the marketing department (Editorial, *CMAJ*, 2002b)

This edit says that for pharma, profit conflicts with (and trumps?) human welfare:

‘No one seriously doubts that pharma’s profit motive is in conflict with its higher human purposes.’

Talking about how business concerns make pharma favour commoner conditions for research sponsorship, it says:

‘It makes business sense for the drug industry to favour common conditions over rarer ones in the search for therapeutic compounds’.

Further, as to why negative findings about new products are kept under wraps or clinical trials are designed to obtain favourable results, it says it also makes for (business) sense:

‘It also makes sense not to shoot a new product in the foot with discouraging data. There is accumulating evidence that companies design clinical trials to obtain results favourable to their product’.

Pharma marketing is likened to a chameleon and advertising becomes subtler even as it becomes bolder:

‘And then there’s that chameleon, marketing. As direct-to-consumer advertising becomes emboldened, messages to clinicians grow more subtle. We have learned to beware the free lunch and trivial giveaway. But what about our acceptance of information that industry has paid for?’

Talking about the inevitability of financial conflict of interest, it says it must be disclosed especially by writers of CPGs (See also Chapter III, p52-53) and reviews, consensus conference participants (See also, the case of Xigris, Chapter III, p45-47) and phase IV Clinical trial recruiters from the medical fraternity:

‘We are not so naive as to imagine that financial conflicts of interest can be eliminated entirely. But they should be disclosed: by physicians who receive money to enrol patients in phase IV trials; by participants in consensus conferences; by the writers of CPGs and reviews.’

And finally talking about how marketing can masquerade as evidence and science, it says:

‘Prescriber beware: marketing departments are experts in disguise and one of those disguises is science.’

### Institutions, contracts and academic freedom (Drazen in *N Engl J Med*, 2002)

Drazen cautions that research contracts with industry are essentially business transactions and that if the peer review system and the scientific process of replication have to help reach the truth, researchers need full access to data and full control over publication:

‘No matter how altruistic the motive, investigators must recognize that research performed under these contracts is a business transaction. It is imperative that the terms of such contracts guarantee the safety and confidentiality of patients while preserving the academic independence of participating investigators. Research performed under a contract that gives the investigators full access to the data and the right to publish their findings, without interference from the sponsor, lets the peer-review system and the scientific process of replication eventually get to the truth.’

Talking next of the results of a study and the naiveté of academia that does not insist on access to primary data and hence exposes itself to a strategy of ‘divide and conquer’, he says:

‘… very few centers included standard language in their contracts that guaranteed the investigators at a given center access to the primary data from the entire study. Without such a guarantee, the entities sponsoring the research can effectively implement a “divide and conquer” strategy that allows each group of investigators access to their own data, but makes analysis of all the data in a multicenter trial a virtual impossibility’.

### Collaborating with industry - choices for the academic medical center (Moses, Braunwald, Martin and Their in *N Engl J Med*, 2002)

Moses *et al* talk of the commercial value of biomedical academic research, strengthening academia-industry bonds and increasing incentives for faculty members to participate in commercialisation:

‘The relationships between academic institutions and private companies are strengthening. The decision of several large pharmaceutical companies and many biotechnology companies, to build major new laboratories near U.S, European and Asian universities is just one example of the growing commercial value of academic innovation in biomedicine and the talent that produces it. Individual faculty members and universities in the United States and other countries have increasingly strong financial and nonfinancial incentives to start new companies and to participate directly in the development of drugs, devices and diagnostic tests’.

While welcoming the connect, the editorial makes some salutary recommendations, especially the arms length approach, keeping IRBs free of economic influence and parity amongst faculty members:

‘Although policies will vary, we think some policies - separation of institutional review boards from economic influences, management of institutional and personal conflicts at arm’s length and consistent treatment of all faculty members, regardless of rank - must be immutable.’

### The next step: ensuring integrity of scientific research (Editorial, *Lancet*, 2002)

This editorial talks of competition for funds in academia, consequent compromise of integrity and the need to retain public trust by scientific excellence that goes beyond personal interests:

‘… the current climate of intense competition for funding and academic positions has created an environment that challenges the concept of personal and sometimes institutional, integrity even further.. only rigorously guarded integrity in research will allow both the achievement of scientific excellence beyond personal gain and retain the public’s trust as new scientific fields are entered.’

### Maintaining the public trust in clinical research (Kelch in *N Engl J Me*d, 2002)

Kelch dwells on the product or patient dilemma due to commercialisation of research:

‘One of the major questions now is how to address potential conflicts of interest or commitment surrounding the commercialization of research - how to strike a balance between the need for investigators to act in the best interests of patients and their desire to serve the interests of the product they are developing.’

What need be done to reduce the inevitable conflicts of interest is full disclosure and an effective peer review process:

‘Conflicts of interest can never be eliminated completely - not in everyday life and not in academic medicine. Those that cannot be eliminated must therefore be recognized, disclosed fully and managed. The extensive peer-review process effectively manages most potential academic conflicts of interest.’

While concluding, in a moment of rare insight, how entrepreneurship must combine with patient care, it says it is not possible to combine it in the same person; which doesn’t mean enterprise is to be abandoned or new therapies discouraged:

‘One cannot work simultaneously as an inventor-entrepreneur and a physician or other health care provider and maintain the trust of patients and the public. To attempt to do so is to challenge the primacy of the doctor-patient covenant. On the other hand, the system must allow enough flexibility for promising new approaches to be tested.’

### Academic freedom in clinical research (Nathan and Weatherall, editorial, in *N Engl J Med* 2002)

These authors caution universities to decide how much to commercialise and how to maintain academic freedom:

‘Universities will have to decide on the extent to which they wish to become commercialized and will have to monitor the effect that such commercialization has on the pattern of their research, on public confidence in research and on academic freedom.’

While optimism is justified, equally important is problems of the academia-industry connect be identified and rectified:

‘We now have the potential to enter one of the most productive periods in biomedical research, the success of which will depend to no small degree on an increasingly close partnership between universities and industry. It is vital, therefore, that the problems of this interface be recognized and corrected.’

We must ensure that there is freedom from fear in academia to express opinions contrary to sponsor interests:

‘All of us in academic medicine must look carefully at our own houses and set standards that protect the rights of faculty members to express their opinions in scholarly settings and journals.’

### Maintaining the integrity of the scientific record (Smith, editorial, *BM*J, 2001)

While justifying the revised ICMJE guidelines as a means to maintain the integrity of the scientific record, Smith expresses the apprehensions of biomedical journal editors who hate to be shortchanged by sponsors getting pliant research published:

‘We editors of medical journals worry that we sometimes publish studies where the declared authors have not participated in the design of the study, had no access to the raw data and had little to do with the interpretation of the data. Instead the sponsors of the study-often pharmaceutical companies have designed the study and analysed and interpreted the data. Readers and editors are thus being deceived. Editors are also concerned that the declared authors might not have ultimate control over whether their studies are published. That decision may rest with the funders of the research perhaps a government department or a pharmaceutical company which could mean that results unfavourable to the funders are suppressed. This distorts the scientific record and again deceives readers, allowing them to read only favourable results.’

### Is the university-industrial complex out of control? (Editorial, *Nature*, 2001)

This editorial encapsulates the concerns voiced by others above when it says that links between academia and industry are of increasing concern to academics and to society at large and the sectors involved must review and revise their policies in order to sustain the public accountability and academic freedom of universities.

## Some Points To Ponder: Paradigm Shift In Attitudes

While discussing trends in thinking of journals, let us study the interesting case of some write-ups in a journal like the *NEJM*. We can see how earlier skepticism and caution sounding of 1994 yields to pragmatism and grudging acceptance by 2002 and even enthusiastic welcome, by 2005.

An earlier editorial in the *NEJM* called *The Journal’s Policy on Cost-Effectiveness Analyses* articulates its stand pretty categorically (Kassirer and Angell, 1994):

… we ask authors of scientific articles who have financial connections with a company that makes the product under study (or its competitors) to inform us of these connections when they submit their manuscripts. We do not make such financial relationships known to reviewers, but we do disclose them to our readers, when appropriate, at the time of publication. In contrast, we do not even consider review articles or editorials by authors with any financial connections to companies whose products are featured prominently in the article (or their competitors).

What is also interesting to note in this connection is the marked change in attitude that has come about in journal policies. Let us take the example of the same *NEJM*. The 1994 editorial is pretty critical of industry-academia relationships, advising to keep them at arms length in relation to cost-effectiveness studies, but applicable to other such situations as well:

Conflicts of interest in clinical research grow more numerous and problematic as the links between academia and industry grow closer and more complex. The likelihood of bias in cost-effectiveness studies can be reduced if these relations are kept at arm’s length (Kassirer and Angell, 1994).

Compare this ‘at arms length’attitude with the relatively recent write-ups in the same *NEJM*, which are cautiously optimistic:

We now have the potential to enter one of the most productive periods in biomedical research, the success of which will depend to no small degree on an increasingly close partnership between universities and industry. It is vital, therefore, that the problems of this interface be recognized and corrected.’(Nathan and Weatherall, 2002).

The relationships between academic institutions and private companies are strengthening (Moses, Braunwald, Martin and Their, 2002).

And a relatively recent one, which is plainly adulatory:

By any measure, the interactions between academic research and industrial research and development, as epitomized by biotechnology, have been overwhelmingly positive. We should celebrate their achievements and protect the process that led to them (Stossel, 2005).

From criticism to caution to optimism to adulation. Attitudes undergo paradigm shifts too.

## Lead Taken By Respected Medical Journals: Revised ICMJE Guidelines

Apart from voicing concern in editorials, some Editors, in September 2001, went ahead and set new rules, the revised ICMJE guidelines. A dozen respected medical Journals like *NEJM, JAMA, Lancet* and *CMAJ* have taken the lead. These journals (Davidoff *et al* 2001) and, following their lead, other journals (Smith, [editorial], BMJ, 2001) now refuse to publish studies unless the responsible author signs a statement that he or she had access to the data, accepts full responsibility for the conduct of the trial and controls the decision to publish:

The journals that are members of the International Committee of Medical Journal Editors, including the BMJ, will now routinely require contributors to disclose details of their own and their funders’roles in the study. We will ask contributors to sign a statement that they accept full responsibility for the conduct of the study, had access to the data and controlled the decision to publish. If authors cannot satisfy us on these points we will not publish. In this way we hope to contribute to maintaining and improving the integrity of the scientific record (Smith, 2001).

A laudatory step indeed, for that ensures, at least on paper, that academia rather than industry has control over publication. That this creates an embarrassing situation for researchers who had not looked into this aspect hitherto and takes the wind off the sails of industry is not anything to gloat over. For be sure some bright minds on both sides would be very busy trying to find ways to bypass the obstacles such embarrassing policing creates. In fact a relatively recent review of industry contracts indicates that most researchers will not be able to meet the new journal requirements unless changes are made (Schulman *et al*, 2002). More of that in the next section.

Even if it were so, it is worth our while to study some features of the revised ICMJE guidelines.

## ICMJE Revised Guidelines

Concerned about threats to the integrity of clinical trials in a research environment increasingly controlled by private interests, the International Committee of Medical Journal Editors (ICMJE) has, in 2001, issued its “Uniform Requirements for Manuscripts Submitted to Biomedical Journals.” (Davidoff *et al* 2001; International Committee of Medical Journal Editors, 2001). In short called URM, these are revised guidelines for investigators’participation in the study design, access to data and control over publication. These revisions call for full disclosure of the sponsor’s role in the research, as well as assurances that the investigators are independent of the sponsor, that investigators be fully accountable for both design and conduct of trials, that they have independent access to all trial data and that they control all decisions related to publication and editorial issues (International Committee of Medical Journal Editors, 2001). In other words, a firm assurance that investigators are independent of sponsors. This is necessary to prevent derailment of biomedical research and its publication to suit sponsors interests. It is also critical to retain public trust, because:

Public trust in the peer review process and the credibility of published articles depend in part on how well conflict of interest is handled during writing, peer review and editorial decision making (Davidoff et al., 2001).

Smith (2001) rightly attempts to allay apprehensions of pharma and concerned researchers when he says:

This initiative by journal editors should not be seen as an attack on the pharmaceutical industry. Almost all new drugs are developed by the industry and many companies have high ethical standards and will see no problem in complying with the new policies. Pharmaceutical companies become successful not through dubious publication or marketing policies but by developing important new drugs.

Indeed. Companies with high ethical standards should have no problems complying with revised ICMJE guidelines. They will however do so only if they realize their future, and success, rests not on doctored publications and questionable marketing techniques, but simply on producing drugs/devices which are significant advances over current ones. Those who are long term players in industry and wish their credibility synergises with profits will neglect such guidelines only to their eventual peril. Fly by night operators or the new entrant-upstarts will of course find such guidelines irksome. It’s important pliant researchers and journals are not allowed to reduce their discomfort. It’s equally important they are not allowed to dictate the agenda. We will come back to this in the last chapter (Chapter X, p121-126).

## Results Of A Study To Determine Adherence To ICMJE Standards

It is one thing to lay down guidelines. It’s another to enforce it or find out if it is followed. For example, Schulman *et al’s* (2002) findings are that it is unclear whether researches conducted at academic institutions adhere to these new standards.

From November 2001 through January 2002, they interviewed officials at U.S. medical schools about provisions in their institutions’agreements with industry sponsors of multicenter clinical trials. Of the 122 medical schools that are member of the Association of American Medical Colleges, 108 participated in the survey. Scores for compliance with a wide range of provisions - from ensuring that authors of reports on multicenter trials have access to all trial data (1 percent [interquartile range, 0 to 21]) to addressing the plan for data collection and monitoring (10 percent [interquartile range, 1 to 50]) - demonstrated limited adherence to the standards embodied in the new ICMJE (Schulman *et al.,* 2002). The conclusion is an eye-opener:

Academic institutions routinely engage in industry-sponsored research that fails to adhere to ICMJE guidelines regarding trial design, access to data and publication rights. Our findings suggest that a reevaluation of the process of contracting for clinical research is urgently needed (Schulman et al., 2002).

In other words, the guidelines maybe great but are not adhered to and the research contracts need a revamp. Is academia listening and can it make industry listen too?

And it’s one thing to lay guidelines, quite another to know if it’s followed and still quite another to know if it’s enforced or enforceable at all.

## Integrity of Industry Sponsored Research

The integrity of industry-sponsored clinical research has come under increasing scrutiny (Schulman et al, 2002). Concerned conscientious observers of the last decade or more have focused on two main issues:

- Investigators’ financial conflicts of interest with industry sponsors (Boyd and Bero, 2000; McCrary *et al,* 2000; Lo, Wolf and Berkeley, 2000; Cho, Shohara, Schissel and Rennie, 2000; DeAngelis, 2000; DeAngelis, Fontanarosa and Flanagin, 2001);
- Publication bias arising from pressure by sponsors to withhold negative research results (Rosenberg, 1996; Blumenthal *et al.,* 1997; Rennie, 1997; [Erratum, 1997]; Dickersin, 1990; Rivara and Cummings, 2002).

The basic shizm is between the value system of a patient welfare driven professional and that of a profit driven industry. While the one wants to avoid control but wants the dough, the other wants to exercise the control by supplying the dough. That’s it, then, in a nutshell.

The key to the whole problem is not sponsorship generated loyality or its expectation. The key is allegiance to patient welfare, research integrity and consequent biomedical advance and both sides reaping legitimate rewards from such allegiance: renewed sponsorship, larger grants, industry profits, heightened credibility *et al*. Any accent otherwise and malevolence has ample scope to insinuate itself, even stymie personal growth and retard biomedical advance itself. It is naïve to believe that one can get away with fooling everyone around for any length of time. Checks and balances become more stringent, opponents become more vociferous and even legitimate advances become suspect. Long-term players with major investments may neglect such realisations to their own peril.

These issues have not remained concerns only on paper. For example in the US, some of these concerns culminated in the publication in 2001 and 2002 of guidelines by the Task Force of the Association of American Medical Colleges for the management of individual and institutional financial interests in biomedical research respectively (Association of American Medical Colleges, 2001 and 2002), something we have detailed separately elsewhere. (See ‘Foundations And Task Forces’, p82-89) In other words, how should individual researchers and institutions regulate themselves in industry-sponsored research. However, recommendations for dealing with conflicts of interest do not address other potential sources of bias in industry-sponsored research, including the role of the sponsor in the study design, investigators’access to data and control over publication (Schulman *et al.*, 2002). These, as anyone will immediately realize, are the real core issues still to be grappled with.

## ICMJE Revised Guidelines Continued

Let us come back to the important development that took place in 2001: joint action taken by an International Committee of Medical Journal Editors (ICMJE). Concerned about threats to the integrity of clinical trials in a research environment increasingly controlled by private interests, the International Committee of Medical Journal Editors (ICMJE) revised its “Uniform Requirements for Manuscripts Submitted to Biomedical Journals: Writing and Editing for Biomedical Publications.” (Schulman *et al.*, 2002; Davidoff *et al.*, 2001; International Committee of Medical Journal Editors, 2001). (It has been further revised in 2004 and 2006. For detailed exposition, see Van Der Wayden’s ICMJE And URM, elsewhere in this monograph: p15-24).

By going beyond revealing financial conflict of interest and publication bias issues because of sponsor’s pressure to withhold negative results, which are important issues in themselves, the ICMJE has taken an important step in ensuring greater accountability in the research process, aborting its publication and possible malevolent impact on gullible readers. A call for full disclosure, not just financial conflict of interest, is as important as the assurance that the investigators are independent of the sponsors, as also the clear understanding and more so, commitment, that the investigators are fully accountable for both the design and conduct of the trial. Also proper is the provision that investigators have independent access to all trial data, which means they control the findings as they are produced and interpreted. Similarly, the independent access is very important, for it ensures they are not ‘guided’in their findings and eventually in reporting, for that results in doctoring of results as well as publication. The provision that it is investigators and not sponsors, who should control publication decisions is also a step in the right direction, for it ensures the responsibility of publication where it should lie - with investigators, rather than sponsors.

### Covert Accountability To Sponsors Not Addressed

All laudable steps indeed. Intended to ensure greater accountability in research. But the issue of the other, covert, accountability is not addressed at all. The accountability of investigators to sponsors, which is necessarily to work in their interests if they do not want funds to dry up, now or in the future. How do we ensure that accountability to research overrides that to sponsors in case of conflict? No sponsor will agree to the ICMJE guidelines, except on paper: which is convenient, to appear clean. It just does not make commercial sense to hand over the reins of publication to the investigators, while the fellows who shell out the money and want to make the profits wait patiently in the sidelines to be offered some crumbs in the form of positive findings of some potential money spinner molecule. One wonders which industry player would play the game thus. The greater chances are the ICMJE guidelines will be followed on paper and royally flouted. And investigators will sell their souls once again in a different form for the proverbial ‘thirty pieces of silver’.

The only good that can result is that, for ethical investigators, there are clear-cut guidelines as to how far they can go and no further. Moreover, for editors and publications dependent of industry funding, the ICMJE has become an important rallying point to take a united stand and spell out how far and how strongly they are ready to go in favour of research even at the risk of antagonizing sponsors. Of course, this does not mean the effort itself may not be a convenient ploy to appear conscientious and carry forward industry’s agenda that much more subtly. But one is circumspect to label a hallowed group like editors as conflicted, like we find it difficult to accept judiciary could be corrupt or the laity still finds it difficult to attribute profit motives to men of medicine. One wonders how far the hallow will hold, given the major influence industry financing will keep having over research journals as time flows by.

One remedy, which suggests itself, but which very few will find acceptable, is to reject industry funding totally, even if it appears genuinely directed, for it is the one major source of potential malfeasance in research. In this connection what Schafer (2004) mentions in the abstract of his paper on the Olivieri and Healy cases is worth a look:

No discussion of academic freedom, research integrity and patient safety could begin with a more disquieting pair of case studies than those of Nancy Olivieri and David Healy. The cumulative impact of the Olivieri and Healy affairs has caused serious self examination within the biomedical research community. The two case studies are. placed in their historical context-that context being the transformation of the norms of science through increasingly close ties between research universities and the corporate world. After a literature survey of the ways in which corporate sponsorship has biased the results of clinical drug trials, two different strategies to mitigate this problem are identified and assessed: a regulatory approach, which focuses on managing risks associated with industry funding of university research and a more radical approach, the sequestration thesis, which counsels the outright elimination of corporate sponsorship. The reformist approach is criticised and the radical approach defended (Schafer, 2004).

This is the obvious conclusion, but if one has the guts to accept it. In its absence, we make norms and guidelines to control the flock, while the sheep are busy feeding on the greener pastures of way layers and enjoying the fare; blissfully unaware of the price they pay in compromising their ethics and their research potential. If nothing else even this much of a realization would be a great achievement of this monograph.

The process of self-correction set into motion due to greater clout of conscientious researchers, unrelenting expose of medical journalists and supportive editors, patients right activism and law suits against industry will, hopefully, help tilt the balance towards value-based advance, even if belated and done grudgingly. The earlier the major players understand this, the better it is for all concerned.

### No Mincing Words And Chills Down Spines

The conclusions of the Schulman *et al* (2002) study also do not mince words:

Our findings suggest that academic institutions routinely participate in clinical research that does not adhere to ICMJE standards of accountability, access to data and control of publication. These standards address long-standing concern about the integrity of research published in biomedical journals. We found that academic institutions rarely ensure that their investigators have full participation in the design of the trials, unimpeded access to trial data and the right to publish their findings (Schulman et al, 2002).

One wonders whether a more categorical indictment of the prevailing state of affairs is possible. The reason mainly listed is, “The current research environment may impede institutions’attempts to negotiate contract provisions that secure investigators’rights” (ibid). Which means, in simple terms, that the sponsors lay down the terms and conditions, not the investigators. What follows makes the tragedy pretty obvious:

In response to some survey items, particularly those addressing publication and confidentiality, several respondents said that they felt powerless in contract negotiations with sponsors. One respondent stated that although some institutions may be able to negotiate provisions that ensure investigators’rights, her institution was ‘just a small medical school.’” (ibid).

What greater proof of who calls the shots in research contracts need be given? Well, the profession is in a real double bind, it has the proverbial Hobson’s choice. It cannot do away with sponsorship and it cannot control it too. So it struggles with provisions and guidelines and ideas of ethics and norms and criteria, while industry has a quiet laugh at the docs’predicament. A quiet laugh that should send chills down some spines at least, if the situation has to be seen with unblinkered eyes and then hopefully remedied.
